# Genome-Wide Analysis of the Metallocarboxypeptidase Inhibitor Family Reveals That *AbMCPI8* Affects Root Development and Tropane Alkaloid Production in *Atropa belladonna*

**DOI:** 10.3390/ijms252413729

**Published:** 2024-12-23

**Authors:** Shengyu Yang, Yi Wang, Shiyu Wan, Can Zhang, Siyuan Liao, Min Chen, Xiaozhong Lan, Zhihua Liao, Lingjiang Zeng

**Affiliations:** 1 Integrative Science Center of Germplasm Creation in Western China (CHONGQING) Science City, SWU-TAAHC Medicinal Plant Joint R&D Centre, School of Life Sciences, Southwest University, Chongqing 400715, China; yangshengyu@email.swu.edu.cn (S.Y.); yiwang1998@email.swu.edu.cn (Y.W.); wsy1201@email.swu.edu.cn (S.W.); zhangcan2004@email.swu.edu.cn (C.Z.); syliao@email.swu.edu.cn (S.L.); zhliao@swu.edu.cn (Z.L.); 2 College of Pharmaceutical Sciences, Southwest University, Chongqing 400715, China; mminchen@swu.edu.cn; 3 TAAHC-SWU Medicinal Plant Joint R&D Centre, Key Laboratory of Tibetan Medicine Resources Conservation and Utilization of Tibet Autonomous Region, Xizang Agriculture and Animal Husbandry University, Nyingchi 860000, China; lanxiaozhong@163.com

**Keywords:** *Atropa belladonna*, metallocarboxypeptidase inhibitor (MCPI), root development, tropane alkaloids

## Abstract

*Atropa belladonna* is a medicinal plant and an important source for the commercial production of tropane alkaloids (TAs), such as scopolamine and hyoscyamine, which are used clinically for their anticholinergic properties. In this study, we identified 16 metallocarboxypeptidase inhibitor (MCPI) genes from *A. belladonna* (*AbMCPIs*), which are grouped into three subgroups based on phylogenetic relationships and are distributed across 10 chromosomes. Promoter analysis showed that most *cis-regulatory* elements were related to defense and stress responses, such as drought, low-temperature, ABA (abscisic acid), GA (gibberellin), auxin, light and MeJA responsiveness. A gene encoding a putative metallocarboxypeptidase inhibitor (*AbMCPI8*) is cloned from *A. belladonna* and characterized. *AbMCPI8* shows similar tissue expression pattern to TA biosynthesis genes such as *AbPMT*, *AbAT4*, *AbTRI*, etc., with exclusive expression in the roots. When *AbMCPI8* is silenced by virus-induced gene silencing (VIGS), the root growth is markedly inhibited and the production of hyoscyamine and scopolamine is significantly reduced. Our findings indicate a positive role of AbMCPI8 in root development, which could positively affect TA production in *A. belladonna*.

## 1. Introduction

Tropane alkaloids (TAs) are a kind of nitrogen-containing secondary metabolite used as a natural drug by humans, which have a long history. They are distributed among diverse plant families, such as Solanaceae, Erythroxylaceae, Euphorbiaceae, Rhizoporaceae and so on [1]. Hyoscyamine and scopolamine are two clinically valuable TAs, which are widely used for treating Parkinsonian tremors, or used as anesthetics or analgesics and antidotes to nerve agents owing to their anticholinergic properties [2]. At present, medicinal plants of the Solanaceae family, such as *Atropa belladonna*, *Datura metel* and alike, are the only natural source available for the commercial production of hyoscyamine and scopolamine [2,3]. Yet, the levels of these TAs in these plants are low, needing to be elevated by metabolic engineering or genetic breeding.

Among these TA-producing plants, *Atropa belladonna* is the most intensively studied one with regard to TA biosynthesis. The hyoscyamine and scopolamine biosynthetic pathway has completely been elucidated, which involves 13 key enzymes, such as putrescine *N*-methyltransferase (PMT) [4], tropinone synthase (CYP82M3) [5], tropine reductase (TRI) [6], aromatic amino acid aminotransferase (AT) [7], littorine mutase (CYP80F1) [8], hyoscyamine 6*β*-hydroxylase (H6H) [9], etc., and originally starts from two amino acid precursors, ornithine and phenylalanine. The clarification of this biosynthetic pathway provides good hope for engineering the production of these TAs.

All the identified key enzyme genes implicated in hyoscyamine and scopolamine biosynthesis are dominantly expressed in roots, especially in secondary roots of these TA-producing plants. TAs biosynthesized in roots are then transferred to aboveground parts, like leaves and flowers of plants, leading to a wide distribution in the various organs [10]. Thus, it is reasonable to speculate that promoting root development to increase its biomass may be conducive to increasing TA contents in these plants. However, there has been no report on the relationship between root development and TA production.

Metallocarboxypeptidases are zinc-dependent exoproteases and play diverse physiological roles [11]. In plants, protease inhibitors are particularly abundant in reproductive and storage organs such as seeds and tubers, and many of them are implicated in defense response against herbivores and pathogens [12,13,14]. Several metallocarboxypeptidase inhibitors (MCPIs), possessing a cystine-knot structure, have been identified in solanaceous plants, such as potato carboxypeptidase inhibitor (PCI) and its homologs TCMP-1/2 from tomato. PCI protein accumulates in potato tubers and in wounded potato leaves and is involved in plant defense against insects [15,16]. TCMP-1 and TCMP-2 are highly expressed in flower buds and fruits, respectively [17], suggesting a role in flower/fruit development. In addition, an MCPI homolog was cloned from the hairy roots of TA-producing *Hyoscyamus niger*, namely HnHR7, which is expressed exclusively in the primordium and base of lateral roots. Overexpression of HnHR7 significantly enhances the frequency of lateral root formation in root cultures of *H. niger*, suggesting a positive role in initiating lateral root formation [18].

In this study, we identified 16 *AbMCPI* genes through genome-wide analysis, and the sequence features, phylogenetic tree, chromosome location, syntenic analysis and gene structure were investigated and conserved sequence analysis, *cis-regulatory* element analysis and gene expression analysis performed based on the genome of *A. belladonna* [19]. To elucidate the function of MCPI genes, we cloned and characterized a putative metallocarboxypeptidase inhibitor gene from *A. belladonna*, named *AbMCPI8*, and identified its role by performing virus-reduced gene silencing (VIGS) in *A. belladonna*. Our results demonstrate that *AbMCPI8* plays a positive role in root development, thus being conducive to TA production in roots. Our findings reveal a link between root development and TA levels, and they provide a candidate gene that may be useful in genetic engineering for cultivating *A. belladonna* lines with a high yield of hyoscyamine or scopolamine.

## 2. Results and Discussion

### 2.1. Identification and Analysis of MCPI Gene Family in A. belladonna

HMMER and BLAST were performed to search the MCPI proteins in the genome of *A. belladonna*. A total of 16 MCPI genes were obtained, named *AbMCPI1* to *AbMCPI16*. The basic physical and chemical properties of the *AbMCPI* proteins such as amino acid number, molecular weight (MW), theoretical isoelectric point (pI), aliphatic index, grand average of hydropathy (GRAVY) and subcellular localizations are summarized in Table 1. The *AbMCPI* proteins contain 72–174 amino acid residues, with molecular weights between 7811.03 and 19,637.28 Da and theoretical isoelectric points between 4.89 and 7.57. Subcellular localization of these proteins showed that eight of them are localized extracellularly, while eight are localized in the chloroplasts.

### 2.2. Phylogenetic Analysis of the MCPI Family

**Figure 1:**
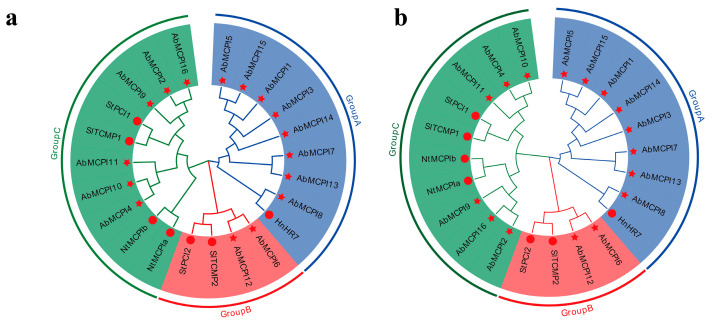
Phylogenetic tree of 23 MCPI proteins from *A. belladonna*, *S. tuberosum*, *S. lycopersicum*, *Nicotiana tabacum* and *Hyoscyamus niger*. (**a**) represents the neighbor-joining tree, (**b**) represents the maximum likelihood tree. The red stars represent the MCPIs of *A. belladonna*, and the red circles represent the MCPIs of other species.

To elucidate the evolutionary relationships among the various MCPI proteins from different Solanaceae plant species, we constructed both neighbor-joining (NJ) and maximum likelihood (ML) phylogenetic trees of the 23 MCPI proteins, including 16 *AbMCPIs* and 5 characterized MCPIs from 4 Solanaceae species. The results showed that the overall structures of the NJ and ML trees were consistent, and both revealed that these MCPI proteins were divided into three groups: group A contained eight *AbMCPI* proteins; group B contained two *AbMCPI* proteins; and group C contained six *AbMCPI* proteins (Figure 1a,b).

### 2.3. Chromosome Location and Syntenic Analysis

**Figure 2:**
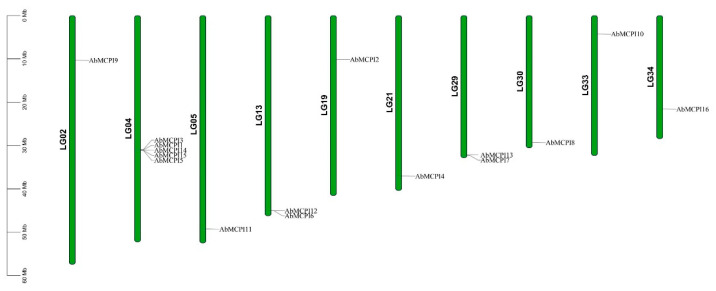
Chromosomal distribution of the *AbMCPIs*.

**Figure 3:**
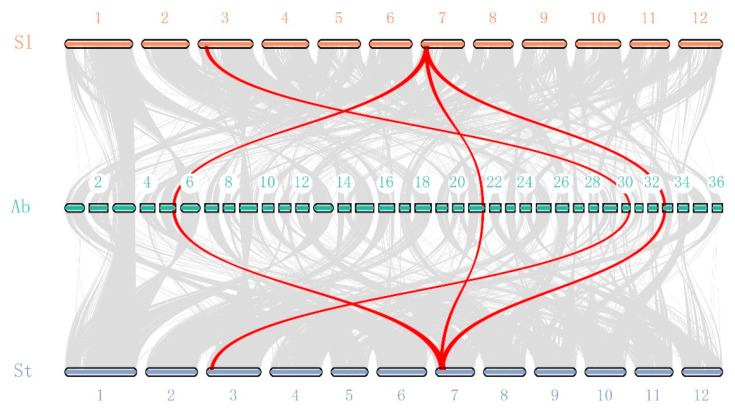
Syntenic relationships of *AbMCPI* genes in *A. belladonna*, *S. lycopersicum* as well as *S. tuberosum*. The numbers represent the chromosome numbers.

To investigate the chromosomal distribution characteristics of the *AbMCPI* family, we analyzed their locations across *A. belladonna* chromosomes. The results showed that 16 *AbMCPI* genes were unevenly distributed in 10 chromosomes in *A. belladonna* (LG02, LG04, LG05, LG13, LG19, LG21, LG29, LG30, LG33, LG34) (Figure 2). Chromosomes LG04, LG13 and LG30 contained four, two and two *AbMCPI* genes, respectively, and chromosome LG02, LG05, LG19, LG21, LG30, LG33 and LG34 contained only one *AbMCPI* gene, respectively. We conducted a syntenic analysis aimed at identifying *AbMCPI* orthologous gene pairs among *A. belladonna*, *S. lycopersicum* and *S. tuberosum*. We detected four orthologous gene pairs between *S. lycopersicum* and *A. belladonna* (*Sl-Ab*) and four orthologous gene pairs between *S. tuberosum* and *A. belladonna* (*St-Ab*) (Figure 3).

### 2.4. Gene Structure and Conserved Sequence Analysis

**Figure 4:**
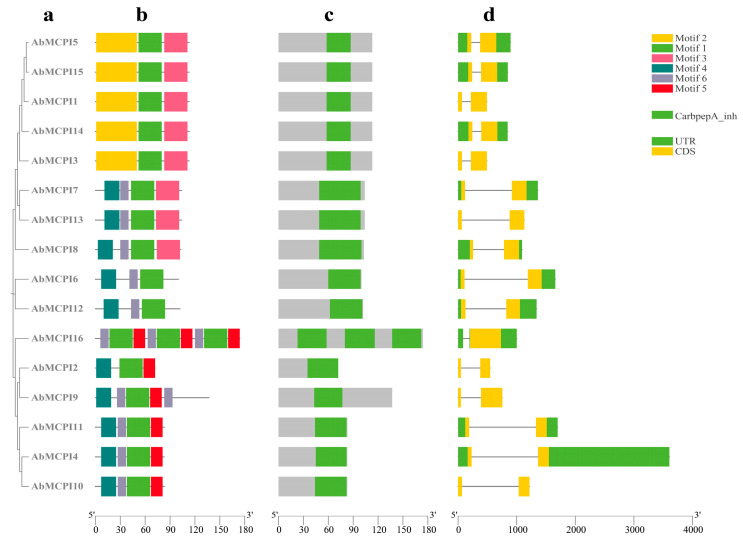
Phylogenetic, multiple motifs, conserved domain and gene structure analysis of the *AbMCPI* family. (**a**) Neighbor-joining (NJ) phylogenetic tree of *AbMCPIs*. (**b**) Motif analysis of *AbMCPIs*. (**c**) Conserved domain analysis of *AbMCPIs*. (**d**) Gene structure analysis of *AbMCPIs*.

We also conducted a phylogenetic analysis with all the identified *AbMCPI* proteins. *AbMCPIs* were clustered as shown in Figure 4a. Motif analysis results suggested that the majority of *AbMCPIs* contained Motif 1, which is specific to the Carboxypeptidase A inhibitor family (Figure 4b). NCBI batch CDD analysis confirmed that each *AbMCPI* sequence contained one conserved Carbpep_inh region (Figure 4c). We investigated the gene structures of *AbMCPIs* and found that 16 *AbMCPI* genes contained one intron (Figure 4d). These results showed that all the *AbMCPIs* exhibited a similar motif composition, indicating that these genes possess general characteristics of the MCPI gene family.

### 2.5. Cis-Regulatory Element and Gene Expression Analysis of AbMCPI Genes in A. belladonna

**Figure 5:**
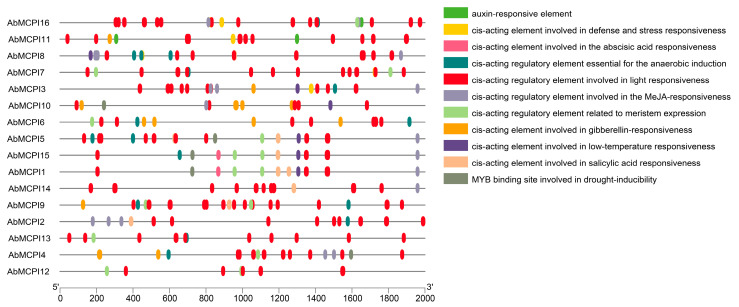
Analysis of *cis-regulatory* elements in *AbMCPIs*.

**Figure 6:**
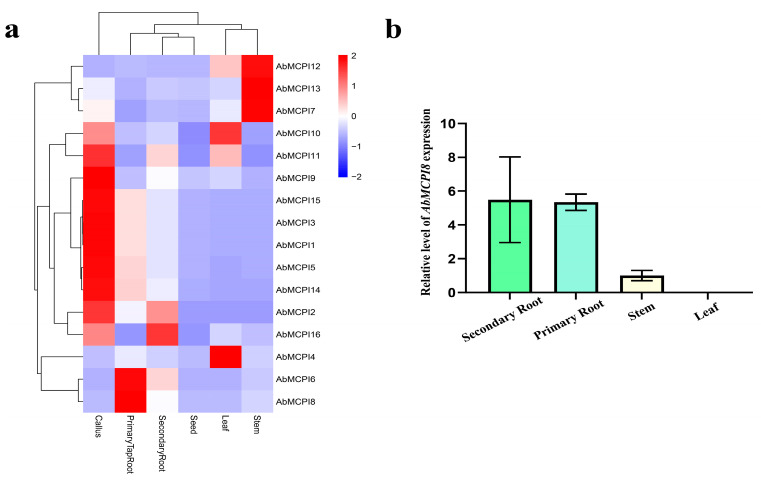
Tissue-specific expression analysis of *AbMCPI* genes in *A. belladonna.* (**a**) Heatmap indicating expression levels of various *AbMCPI* genes in different tissues; (**b**) qPCR analysis of *AbMCPI8* expression level in different tissues. The bars denote means ± standard deviations (n ≥ 3).

To investigate the regulation of *AbMCPI* genes, we analyzed the *cis-regulatory* elements in the promoters of the 16 *AbMCPIs* in *A. belladonna*. Several elements associated with defense and the stress response, such as those responsive to drought, low-temperature, ABA (abscisic acid), GA (gibberellin), auxin, light and MeJA, were identified (Figure 5). These results suggested that *AbMCPIs* are potentially involved in regulating responses to various biotic and abiotic stresses, but further research is needed to validate the definite roles of these elements. Gene expression analysis showed that the *AbMCPI* genes were expressed in different tissues (Figure 6a): *AbMCPI7*, *AbMCPI12* and *AbMCPI13* were markedly expressed in the stem; *AbMCPI4* and *AbMCPI10* were significantly expressed in the leaves; *AbMCPI16* was significantly expressed in secondary roots; *AbMCPI1*, *AbMCPI2*, *AbMCPI3*, *AbMCPI5*, *AbMCPI9*, *AbMCPI14* and *AbMCPI15* were significantly expressed in the callus; and *AbMCPI6* and *AbMCPI8* were markedly expressed in the primary root. Furthermore, tissue profile analysis by qPCR showed that *AbMCPI8* is mainly expressed in primary and secondary roots, while it is expressed at a much lower level in aboveground parts like stems or leaves (Figure 6b).

### 2.6. Molecular Cloning and Sequence Analysis of AbMCPI8 Gene

*A. belladonna* and *H. niger* can produce tropane alkaloids (TAs). Based on the phylogenetic tree and RNA-seq analysis, we selected the *AbMCPI8* gene, which clusters with *HnHR7* in the phylogenetic tree (Figure 1), for cloning and subsequent experimental analysis to investigate its effect on root development and tropane alkaloid production in *A. belladonna*. Based on the sequenced *A. belladonna* transcriptome, the coding sequence of *AbMCPI8* was obtained (Figure S1a). There are five Cys residues scattered from residues 68 to 87 in its protein sequence, which form three disulfide bonds that are thought to play an important part in the direct interaction between the inhibitor and carboxypeptidase [11]. Multiple alignments showed that the protein sequence of *AbMCPI8* is homologous to MCPIs from other plant species, especially with an identity of 88.3% to that of root-specific HnHR7 from *H. niger* (Figure S1b).

### 2.7. Effects of Silencing AbMCPI8 on Root Development and Biosynthesis of Tropane Alkaloids

**Figure 7:**
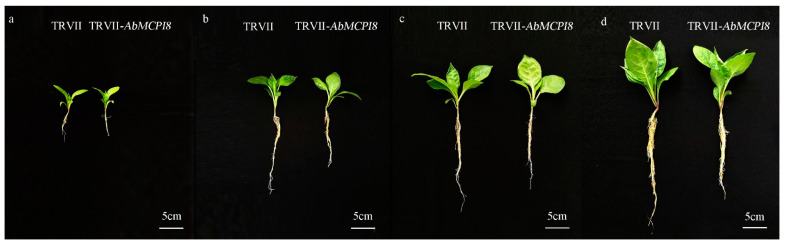
Comparison between the phenotypes of *AbMCPI8*-silencing (TRVII-*AbMCPI8*) and control (TRVII) *A. belladonna* lines at 10 days (**a**), 17 days (**b**), 21 days (**c**) and 25 days (**d**) after *A. tumefaciens*-mediated transformation.

**Figure 8:**
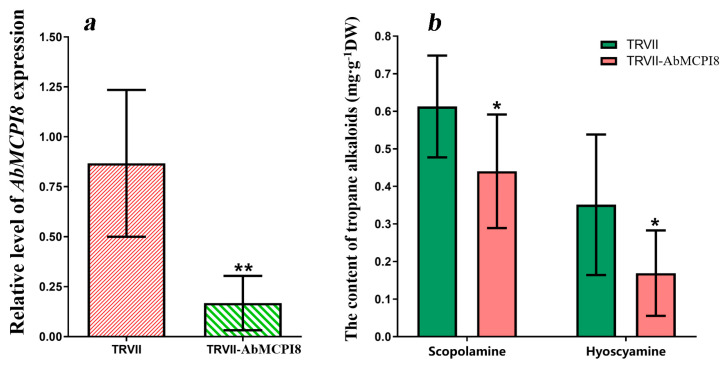
*AbMCPI8* expression levels (**a**) and TA contents (**b**) in *AbMCPI8*-silencing and control plants, respectively. The bars denote means ± standard deviations (n ≥ 3). * and ** indicate significant differences from the control at the levels of *p* < 0.05 and *p* < 0.01, respectively, as determined by the *t*-test.

VIGS technology is a useful approach to characterize the function of genes in plants, which has been demonstrated to be quite effective in the Solanaceae family [20]. We transiently silenced the *AbMCPI8* gene in *A. belladonna* by using VIGS technology 15 d after germination to figure out whether *AbMCPI8* influences root development. When comparing the root development between control lines (TRVII) and *AbMCPI8*-silenced lines (TRVII-*AbMCPI8*), we found that the root growth was significantly reduced after *AbMCPI8* was silenced (Figure 7). Q-PCR results showed that the expression of *AbMCPI8* was significantly decreased in TRVII-*AbMCPI8* transgenic lines when compared with that in the control (transformed with the TRVII empty vector) (Figure 8a). The VIGS results suggest that *AbMCPI8* plays a positive role in root development.

As tropane alkaloids (TAs) such as scopolamine and hyoscyamine are mainly biosynthesized in roots, we infer that silencing of *AbMCPI8* would have an effect on TAs’ biosynthesis by affecting root development. So, the contents of TAs, including scopolamine and hyoscyamine, were detected by HPLC. The *AbMCPI8*-silenced (TRVII-*AbMCPI8*) plants produced hyoscyamine and scopolamine at levels of around 0.17 mg/g dry weight (DW) and 0.44 mg/g DW, respectively, while the control plants produced hyoscyamine and scopolamine at levels of around 0.35 mg/g DW and 0.61 mg/g DW, respectively (Figure 8b). Thus, the silencing of *AbMCPI8* led to a reduction in hyoscyamine and scopolamine contents in *A. belladonna*. The above results suggested that *AbMCPI8* plays a positive role in the regulation of root growth in *A. belladonna*, which consequently has a positive influence on TAs’ biosynthesis.

## 3. Materials and Methods

### 3.1. Identification of the MCPI Family in Atropa belladonna

To identify the members of the MCPI family in *A. belladonna*, we downloaded the genome and annotation file of *A. belladonna* from the Genome Warehouse of the National Genomics Data Center under accession GWHBOWM00000000. The Hidden Markov Model (HMM) file of PF02977 was downloaded from the Pfam database (http://pfam-legacy.xfam.org (12 September 2024)). The identification of MCPI gene family members was performed using the HMM method with an E-value threshold of 0.001 on HMMER 3.2 [21] software. In addition, we downloaded the MCPI protein sequences of *Solanum tuberosum* (AAC95130, NP_001275375) and *S. lycopersicum* (NP_001233934, NP_001234762) from the NCBI database (https://www.ncbi.nlm.nih.gov (12 September 2024)) and used BLAST (2.6.0) [22] software to align the determined MCPI gene family members in the *A. belladonna* genome. We integrated the results of HMMER and BLAST to obtain candidate genes. Following this, candidates were verified through the Pfam database search and NCBI Batch CD-search. The molecular weight (MW) and isoelectric point (PI) of MCPI proteins were calculated using the online ExPASy website (https://web.expasy.org/protparam (13 September 2024)). WoLF PSORT online web (https://wolfpsort.hgc.jp/ (13 September 2024)) was used for subcellular localization prediction of MCPI protein in *A. belladonna*.

### 3.2. Phylogenetic Analysis of the MCPI Family

We performed a sequence alignment of the full-length protein sequences of 23 MCPIs from diverse plant species (*A. belladonna*, *S. tuberosum*, *S. lycopersicum*, *Nicotiana tabacum* and *Hyoscyamus niger*) using the default parameters in the ClustalW program in MEGAX [23] software. We constructed phylogenetic trees using both the neighbor-joining (NJ) method and the maximum likelihood (ML) method in MEGAX, with 1000 bootstrap replicates.

### 3.3. Chromosome Location and Syntenic Analysis of the MCPI Family

The chromosome location information of *AbMCPI* family members was extracted from the *A. belladonna* genome annotation file. TBtools [24] software (2.142) was used to visualize the chromosomal distribution of the *AbMCPI* gene. We conducted a syntenic analysis aimed at identifying *AbMCPI* orthologous gene pairs between *A. belladonna* and *S. lycopersicum* as well as *S. tuberosum* using a Multiple Collinearity Scan Toolkit (MCScanX) [25].

### 3.4. Gene Structure and Conserved Motif Analysis

The positions of exons and introns of each *AbMCPI* gene were obtained from the genome annotation file of *A. belladonna*. Conserved motifs in the *AbMCPI* proteins were analyzed by MEME suite (http://meme-suite.org/tools/meme (13 September 2024)) with the following parameters: search time = 14,400 s, maximum number of motifs = 6, and optimum motif width ranged from 6 to 50 bp. The motif corresponding to the pfam domain was indicated by the Pfam database. The gene structures and protein motifs were visualized by TBtools software (2.142).

### 3.5. Analysis of Upstream Cis-Regulatory Element of MCPI Gene in A. belladonna

The 2000 bp DNA sequence upstream of the MCPI genes was extracted from the genome of *A. belladonna* as the promoter sequence, and its *cis-regulatory* elements were predicted using PlantCARE (https://bioinformatics.psb.ugent.be/webtools/plantcare/html/ (13 September 2024)). The results were visualized using TBtools software.

### 3.6. Tissue Expression by RNA-Seq

Tissue expression analysis was performed using the RNA-seq data obtained from our previous study [19]. The RNA-seq data were normalized using TPM (transcripts per million) values to ensure accurate and comparable expression levels across different tissues. The heatmap of *AbMCPI* expression was visualized using R software package “pheatmap” (4.2.0).

### 3.7. Sequence Cloning and Analysis

Total RNA was extracted from different organs of *A. belladonna* using the plant RNA extraction kit according to the manufacturer’s protocols (Tiangen Biotech, Peking, China). RNA was then reverse transcribed into cDNA as the template for PCR cloning or quantitative PCR (q-PCR) analysis. A pair of gene-specific primers, AbMCPI8-CDS-F/-R (Supplementary Table S1), were used to clone the *AbMCPI8* coding sequence according to the *A. belladonna* transcriptome data. In addition, we cloned the 3′-UTR of *AbMCPI8* using the primers AbMCPI8-RACE-3′-1/-2 by applying RACE technology. The sequences of primers used here are listed in Supplementary Table S1. Multiple alignments among MCPIs from different plant species were carried out with the ClustalW program in MEGAX.

### 3.8. RNA Extraction and RT-qPCR Analysis

*A*. *belladonna* plants were grown in the plant garden of Southwest University. Once the *A. belladonna* plants reached 20 cm in height, different organs, including secondary roots, primary roots, stems and leaves, were collected for RNA isolation. Real-time quantitative PCR (q-PCR) analysis was conducted to analyze relative expression levels of target genes in different organs or in different lines. Q-PCR was performed on an IQ™5 thermocycler (Bio-Rad, Hercules, CA, USA). The *PGK* gene of *A. belladonna* was used as the internal reference gene [26]. The primers used in q-PCR analysis, PGK-Q-F/-R and AbMCPI-Q-F/-R, are listed in Supplementary Table S1. Three biological repeats were measured for each sample.

### 3.9. Silencing of AbMCPI8 in Atropa belladonna Plants

The tobacco rattle virus vector (pTRVII) was used for virus-induced gene silencing (VIGS). A fragment of *AbMCPI8* was amplified using a pair of primers, AbMCPI8-VIGS-F/-R containing *Xho* I and *Kpn* I restriction sites, respectively (Supplementary Table S1), and then ligated into the pTRVII vector to generate the TRVII-*AbMCPI8* vector silencing the expression of *AbMCPI8*. In addition, we also constructed the TRVII-*AbPDS* vector in the same way as above to silence the expression of phytoene desaturase gene (*AbPDS*), which would bleach the leaves of *A. belladonna* as a sign to show whether the VIGS system was working normally. Both the TRVII-*AbMCPI8* and TRVII-*AbPDS* plasmids for VIGS were introduced into the *Agrobacterium tumefaciens* strain GV3101 for plant transformation. *A. belladonna* plants transformed with the pTRVII empty vector were used as the control lines. One month after infiltration by GV3101 strains, the leaf and root materials from transgenic and control *A. belladonna* lines were harvested for further analysis.

### 3.10. Tropane Alkaloid Analysis

Freeze-dried root and leaf materials were ground into powder and used for tropane alkaloids’ extraction. The extraction of the TAs was based on the previously reported method with some modifications [27]. In general, 200 mg dry powder was put into 1 mL extraction buffer (20% methanol, including 0.1% formic acid) and shaken for 2 h at 200 rpm (25 °C). Then, the extract was filtered through a 0.22 μm Nylon66 filter, and the filtrate was diluted 50-fold for analysis. Two types of TAs, hyoscyamine and scopolamine, were detected by HPLC (LC-20AD, Shimadzu, Kyoto, Japan). The detection wavelength was 226 nm. The temperature of the column (150 mm × 4.6 mm) was 40 °C. The mobile phase was composed of 11% acetonitrile and 89% buffer solution (20 mM ammonium acetate and 0.1% formic acid, pH 4.0). The speed of flow was 1 mL/min, and the injected sample solution was 20 μL each time.

## 4. Conclusions

In this study, a metallocarboxypeptidase inhibitor gene, *AbMCPI8*, was cloned from *A. belladonna*. It shows a similar expression pattern to key enzyme genes in TA biosynthesis, such as *AbH6H*, *PMT*, *TRI* and so on, which are specifically expressed in the roots. The VIGS experiment indicated a positive regulatory role of *AbMCPI8* in root development of *A. belladonna*, which has a positive influence on TA biosynthesis. Our study reveals a link between root development and TAs’ levels, and it provides a candidate gene that may be used to improve root growth, to increase TA production in *A. belladonna*.

## Figures and Tables

**Table 1:** Detailed information of the MCPIs in *A. belladonna*.

mRNA ID	Gene Name	Chr	Number (aa)	MW (Da)	pI	Aliphatic Index	GRAVY	Subcellular Location
EVM0064594.1	*AbMCPI1*	LG04	113	12,100.89	5.59	92.39	0.241	chloroplast
EVM0059786.2	*AbMCPI2*	LG19	72	7811.03	6.09	65.14	−0.151	extracellular
EVM0058529.1	*AbMCPI3*	LG04	113	12,158.93	5.10	91.50	0.194	chloroplast
EVM0055767.1	*AbMCPI4*	LG21	83	8953.52	4.95	84.58	0.431	extracellular
EVM0046862.2	*AbMCPI5*	LG04	113	12,100.89	5.59	92.39	0.241	chloroplast
EVM0042929.2	*AbMCPI6*	LG13	100	11,311.24	6.93	77.10	0.115	extracellular
EVM0037680.2	*AbMCPI7*	LG29	104	11,399.30	6.51	110.67	0.393	extracellular
EVM0035594.3	*AbMCPI8*	LG30	103	11,376.30	7.53	96.60	0.221	chloroplast
EVM0032194.2	*AbMCPI9*	LG02	137	15,014.40	5.59	93.28	0.047	extracellular
EVM0030066.1	*AbMCPI10*	LG33	83	9012.59	5.38	81.08	0.339	extracellular
EVM0024721.1	*AbMCPI11*	LG05	83	8906.50	6.00	84.70	0.401	extracellular
EVM0022791.2	*AbMCPI12*	LG13	102	11,559.57	5.88	88.04	0.126	extracellular
EVM0018358.1	*AbMCPI13*	LG29	104	11,326.25	7.57	107.79	0.388	chloroplast
EVM0018026.1	*AbMCPI14*	LG04	113	12,086.87	5.59	92.39	0.240	chloroplast
EVM0007668.2	*AbMCPI15*	LG04	113	12,100.89	5.59	92.39	0.241	chloroplast
EVM0002213.2	*AbMCPI16*	LG34	174	19,637.28	4.89	52.70	−0.564	chloroplast

## Data Availability

The genome and transcriptome data for *Atropa belladonna* are sourced from the research group’s previous studies with accession number PRJNA766188. Further inquiries can be directed to the corresponding author.

## Supplementary Materials

The following supporting information can be downloaded at: https://www.mdpi.com/article/10.3390/ijms252413729/s1.
