# Supplementary material for: Genome-Wide Analysis of the Metallocarboxypeptidase Inhibitor Family Reveals That AbMCPI8 Affects Root Development and Tropane Alkaloid Production in Atropa belladonna
Source: Int J Mol Sci. 2024 Dec 23;25(24):13729. doi: 10.3390/ijms252413729 (PMC11676366; doi:10.3390/ijms252413729)
Supplement: Supplementary file 1 [file ijms-25-13729-s001.zip › ijms-3323255-supplementary.pdf]

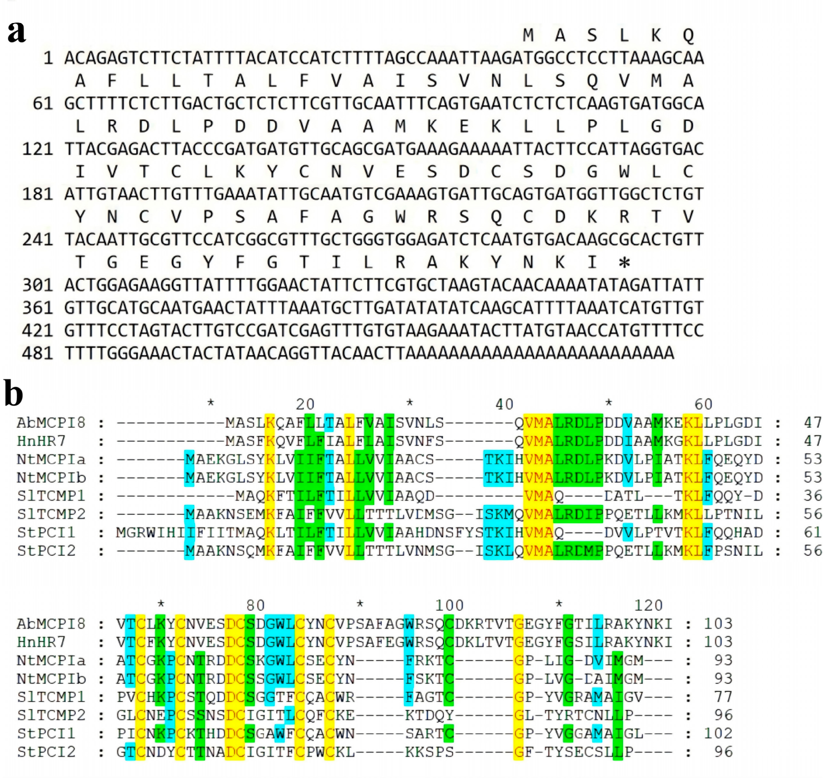

Supplementary Figure S1. Coding sequence of *AbMCPI8* and multiple alignments of plant MCPIs. a. The coding region and translated amino acid sequence of *AbMCPI8*; b. Multiple alignments of MCPIs from several different plant species. *HnHR7*, *NtMCPIa*, *NtMCPIb*, *SlTCMP1*, *SlTCMP2*, *StPCI1* and *StPCI2* represent MCPI sequences from *H. niger*, *Nicotiana tabacum*, *S. lycopersicum* and *S. tuberosum* respectively, which are accessible from the NCBI database.

Supplementary Table S1. Primers used for gene expression analysis in this study.

Primer	Sequence (5\`-3\`)
AbMCPI-CDS-F	ACAGAGTCTTCTATTTTACATCC
AbMCPI-CDS-R	GTTCATTGCATGCAACAATAATC
AbMCPI-RACE-3\`-1	TCTTCGTGCTAAGTACAACAAAAT
AbMCPI-RACE-3\`-2	GCATGCAATGAACTATTTAAATGC
AbMCPI-VIGS-F	cgcCTCGAGGATGAAAGAAAAATTACTTC
AbMCPI-VIGS-R	cgcGGTACCAGTTGTAACCTGTTATAGTA
PGK-Q-F	CAGATACCGTCCTAGTCTCAAC
PGK-Q-R	CAGCCTTGCGACCATACTC
AbMCPI-Q-F	TGATGGCATTACGAGACT
AbMCPI-Q-R	AACAAGTTACAATGTCACCTAA
